# Supplementary material for: OpenMM 7: Rapid development of high performance algorithms for molecular dynamics
Source: PLoS Comput Biol. 2017 Jul 26;13(7):e1005659. doi: 10.1371/journal.pcbi.1005659 (PMC5549999; doi:10.1371/journal.pcbi.1005659)
Supplement: S1 Source Code — (ZIP) [file pcbi.1005659.s002.zip › OpenMM-7.0.1-Source/docs/api-c++/generated/OpenMM.Continuous1DFunction.html]

Continuous1DFunction — OpenMM @OPENMM\_MAJOR\_VERSION@.@OPENMM\_MINOR\_VERSION@ documentation


# Continuous1DFunction¶

*class* `OpenMM::``Continuous1DFunction`¶
:   This is a `TabulatedFunction` that computes a continuous one dimensional function.

    Methods

    |  |  |
    | --- | --- |
    | `Continuous1DFunction` | Create a `Continuous1DFunction` f(x) based on a set of tabulated values. |
    | `getFunctionParameters` | Get the parameters for the tabulated function. |
    | `setFunctionParameters` | Set the parameters for the tabulated function. |
    | `Copy` | Create a deep copy of the tabulated function. |

    `Continuous1DFunction`(*const* std::vector<double> &*values*, double *min*, double *max*)¶
    :   Create a `Continuous1DFunction` f(x) based on a set of tabulated values.

        |  |  |
        | --- | --- |
        | Parameters: |  |

        - **values** – the tabulated values of the function f(x) at uniformly spaced values of x between min and max. A natural cubic spline is used to interpolate between the tabulated values. The function is assumed to be zero for x < min or x > max.
        - **min** – the value of x corresponding to the first element of values
        - **max** – the value of x corresponding to the last element of values

    void `getFunctionParameters`(std::vector<double> &*values*, double &*min*, double &*max*) *const*¶
    :   Get the parameters for the tabulated function.

        |  |  |
        | --- | --- |
        | Parameters: |  |

        - **values** – [out] the tabulated values of the function f(x) at uniformly spaced values of x between min and max. A natural cubic spline is used to interpolate between the tabulated values. The function is assumed to be zero for x < min or x > max.
        - **min** – [out] the value of x corresponding to the first element of values
        - **max** – [out] the value of x corresponding to the last element of values

    void `setFunctionParameters`(*const* std::vector<double> &*values*, double *min*, double *max*)¶
    :   Set the parameters for the tabulated function.

        |  |  |
        | --- | --- |
        | Parameters: |  |

        - **values** – the tabulated values of the function f(x) at uniformly spaced values of x between min and max. A natural cubic spline is used to interpolate between the tabulated values. The function is assumed to be zero for x < min or x > max.
        - **min** – the value of x corresponding to the first element of values
        - **max** – the value of x corresponding to the last element of values

    Continuous1DFunction \*`Copy`() *const*¶
    :   Create a deep copy of the tabulated function.

High performance molecular simulation on GPUs


### Navigation

- Core Objects
- Forces
- Integrators
- Extras

©2015, Stanford University and the Authors.
|
Powered by Sphinx 1.3.1
& Alabaster 0.7.7
|
Page source
